# A Systematic Review and Narrative Synthesis of Interventions for Parental Human Immunodeficiency Virus Disclosure

**DOI:** 10.3389/fpubh.2017.00187

**Published:** 2017-08-07

**Authors:** Donaldson F. Conserve, Michelle Teti, Grace Shin, Juliet Iwelunmor, Lara Handler, Suzanne Maman

**Affiliations:** ^1^Department of Health Promotion, Education, and Behavior, Arnold School of Public Health, University of South Carolina, Columbia, SC, United States; ^2^Department of Health Sciences, University of Missouri, Columbia, MO, United States; ^3^Department of Health Behavior, Gillings School of Global Public Health, University of North Carolina at Chapel Hill, Chapel Hill, NC, United States; ^4^Department of Kinesiology and Community Health, University of Illinois at Urbana-Champaign, Champaign, IL, United States; ^5^Health Sciences Library, University of North Carolina at Chapel Hill, Chapel Hill, NC, United States

**Keywords:** parental human immunodeficiency virus disclosure, interventions, systematic review, human immunodeficiency virus-affected families, children

## Abstract

**Introduction:**

Disclosure of parental human immunodeficiency virus (HIV) infection to their children remains a difficult process for parents living with HIV (PLWH). In order to identify the best strategies to facilitate parental HIV disclosure, it is necessary to examine the efficacy of existing interventions designed to help PLWH parents with the disclosure process to their children.

**Objectives:**

To systematically review the efficacy of interventions designed to assist PLWH disclose their HIV status to their children.

**Methods:**

We conducted a systematic review and narrative synthesis of interventions designed to assist PLWH disclose their HIV status to their children. MEDLINE/PubMed, PsycINFO, Embase, Global Health, and Web of Science were searched.

**Results:**

Studies were eligible for inclusion if they evaluated an intervention for parental HIV disclosure. Five studies published between 2001 and 2015 met the inclusion criteria. The interventions were conducted in South Africa, China, and the United States. Three of the studies used two-arm randomized controlled trials, in which the intervention group was given enhanced care while the control group received standard care. Four of the five studies included a theoretically informed intervention and three were limited to mothers. Results showed that four of the interventions increased parental HIV disclosure.

**Conclusion:**

The findings suggest that parental HIV disclosure interventions are successful in assisting parents with the disclosure process and can be adapted in different cultural context. Future parental HIV disclosure interventions should include fathers in order to assist men with parental HIV disclosure.

## Introduction

Disclosure of parental human immunodeficiency virus (HIV) infection to children remains a difficult process for families affected by HIV. In fact, parents living with HIV (PLWH) describe disclosure as one of their greatest challenges (1). Research indicates that anywhere from 34% to over 80% of PLWH have not disclosed their HIV status to their children (2) and that they often struggle to figure out when and how to disclose to their children (3–7). Many parents, for example, do not disclose to their children for a number of reasons including children’s lack of cognitive-developmental ability to understand the illness (1, 8–10).

Although the World Health Organization Guidelines on Disclosure to Children suggest that school age (i.e., 6–12 years) children can potentially understand and cope with their parent’s illness (11), numerous disclosure barriers exist. For example, some parents report that they do not disclose their HIV status to their children because they lack self-efficacy and simply do not know how to disclose (12–15). Others indicate that they do not disclose due to fear of children’s inadvertent disclosure of parents’ HIV-positive status to others in the community and the ensuing stigma and discrimination children and parents may face (16–18). For some, disclosure of sero-positive status requires an understanding of the sociocultural factors that shape individual and collective decisions surrounding health and well-being. Sociocultural factors which refer to the factors that represent the collective consciousness of people, active enough to influence and condition perception, judgment, communication, and behavior have been found to influence the taken-for-granted assumptions about identity and issues of belonging with parents’ fear of rejection and loss of respect from their children, for example, serving as barriers to parental HIV disclosure (14, 19–21). Still, others cite concerns about causing their children emotional pain and psychological harm (15, 22).

To date, the literature on the psychological effects of parental HIV disclosure on children is mixed (1). Some researchers have reported that there is no relationship between parental disclosure and child functioning (23–25). Other studies have found that children who are aware of their parents’ HIV status have lower social and emotional functioning (26), greater mental distress (24), and more externalizing symptoms (16). The amount of information children receive about their parents’ illness during the disclosure process may influence the disclosure outcome, particularly the psychological impact on children. For example, parents who inform their children that they have HIV but that they are also taking antiretroviral medicines can effectively reassure children that the medication will improve their health, thereby reducing the child’s worry about their parent’s health (27). In contrast, parental HIV disclosure without further explanation about treatments may lead to fear and anxiety that can impair children’s psychological functioning (26).

Based on the challenges HIV-positive parents face with the disclosure process to their children regarding when and how to disclose to their children, and the possible negative consequences of disclosure, more interventions are needed to assist PLWH who want to inform their children about their HIV status, especially in low-and-middle income countries (28). In order to identify the best strategies to facilitate parental HIV disclosure, it is necessary to examine the efficacy of the existing interventions designed to help PLWH parents with the disclosure process to their children. Although a number of review papers have been published describing the factors that influence parental disclosure, their focus was not on the effectiveness of existing interventions (1, 2, 29–34). We aim to fill an important gap in the literature by conducting a systematic review and narrative synthesis of interventions designed to promote parental HIV disclosure to children.

## Methods

### Search Strategy

A literature search was conducted in May 2016 for papers that met the inclusion criteria. The electronic search included MEDLINE/PubMed, PsycINFO, Embase, Global Health, and Web of Science. These databases were selected to cover a wide range of disciplines, from social sciences to interdisciplinary and medical research. A combination of controlled vocabulary and Boolean-paired keywords were used, relating to acquired immunodeficiency syndrome (AIDS), HIV, disclosure, parents or children, and interventions (Table 1). In addition to searching electronic databases, the authors also reviewed the bibliographies of selected studies for other relevant citations.

**Table 1:** Search strategy for MEDLINE/PubMed.

Search category	Acquired immunodeficiency syndrome/human immunodeficiency virus	Disclosure	Children/parents	Interventions
Search terms	(“acquired immunodeficiency syndrome” OR HIV)	(Disclosure OR tell OR talk OR told)	(Children OR child OR mothers OR fathers OR parents)	(Intervention OR interventions)

### Inclusion Criteria

Research studies that met the following criteria were included: (i) the paper discussed disclosure of HIV status by parents to their children, (ii) the study population included HIV-infected parents, (iii) an intervention was evaluated, and (iv) the paper was published in English. Exclusion criteria included non-parental HIV infection disclosure intervention including conference abstracts or dissertations, and papers written in languages other than English. There were no limits for study location or publication date.

### Full Text Review

All articles were initially screened by two reviewers who independently reviewed the titles and abstracts of studies to accept or reject for full text review. The same two reviewers independently reviewed the full texts of the studies identified from the electronic search to determine if they were still eligible to undergo data extraction. In order to be included, studies had to evaluate an intervention designed to promote parental HIV disclosure to their children. Data were extracted from eligible studies into an electronic spreadsheet. Reviewers discussed any disagreements in the data extracted, and referral to a third reviewer was done to resolve any disputes. We extracted the following data: study characteristics (author, sample, study design, comparison/control components, intervention components, assessment, outcome variable, and outcomes) (Table 2). Finally, we conducted a narrative synthesis of studies meeting the inclusion criteria. Narrative synthesis which is “*an approach to the systematic review and synthesis of findings from multiple sources and relies primarily on the use of words and text to summarize and explain the findings of the synthesis*” (35). It is used when statistical meta-analysis or another specialist form of synthesis (such as meta-ethnography for qualitative studies) is not feasible particularly due to substantial methodological and clinical heterogeneity between studies identified (35).

**Table 2:** Study and intervention characteristics of the five studies.

Reference	Sample characteristics	Study design	Comparison/control components	Intervention components	Assessment (compensation paid)	Outcome variable	Outcomes
Rotheram-Borus et al. (36)	Parents with acquired immunodeficiency syndrome (*n* = 307) and their adolescent children (*n* = 412) in New York City	Randomized controlled trial (RCT)	Control condition (parents: *n* = 154, youths: *n* = 207) received standard care	Intervention group (IG) (parents: *n* = 126, youths = 118; of 153 parents, 27 were ineligible and of 205 youths, 87 were ineligible) received an intensive intervention. The intervention was delivered in 2 modules, the first module to parents alone (4 Saturdays) and the second module to both parents and adolescents (8 Saturdays). In module 1, parents made decisions regarding disclosure. In module 2, each Saturday involved some time with parents meeting alone while their children met in separate groups, along with sometime during which parents and youths were together in groups. Two sessions were held each Saturday, one 2-h session in the morning and another 2-h session in the afternoon (after lunch). The design of the intervention was based on social learning principles	Parents and adolescents were assessed in individual interviews at 3-month intervals over 24 months, and subjects received $25 for each interview ($50 for parent and youth assessment)	Presence (1) or absence (0) of human immunodeficiency virus (HIV) disclosure to each adolescent and to all adolescents in the family was calculated	There were no significant differences in disclosure or custody plans across conditions, as the completion of repeated interviews regarding disclosure and custody plans represents a significant intervention in itself, and families in both conditions experienced these assessments

Murphy et al. (37)	80 mothers living with HIV and child dyads in Los Angeles, United States	RCT	Control condition (*n* = 41) received standard care (e.g., medical care and case management)	IG (*n* = 39) received three-session intervention in addition to standard care. Intervention sessions addressed children’s typical development, pros and cons of disclosure, mother–child communication, and behavioral practice for disclosure	Baseline, 3, 6, and 9 months. After each completed appointment, mothers were paid $45 for each interview and $45 for each intervention session, and children were allowed to select a toy or toys worth approximately $25 or a retail gift card	At each follow-up, the mother was asked whether she had disclosed to the child that she is HIV+. If the mother had not disclosed, the above baseline information was collected again. If the mother had disclosed, information was collected on the disclosure, including the date of the disclosure, the child’s reactions to the disclosure, and how the mother felt she handled the disclosure	MLHs in the IG were 6 times more likely to disclose their HIV status than those in the control group (OR = 6.33, 95% CI: 1.64, 24.45), with 33% disclosing in the IG compared with 7.3% in the control group. MLHs in the IG showed increases in disclosure self-efficacy across time, increased communication with their child, and improvement in emotional functioning. Children of MLHs in the IG exhibited reductions in depression and anxiety, and increases in happiness

Rochat et al. (38)	24 Zulu families in rural South Africa (within the Africa Centre Demographic Surveillance Area); all mothers were HIV-positive and had an HIV-negative child aged 6–9 years	Uncontrolled pre- and post-intervention evaluation	Each mother served as her own control	Lay counselors delivered the six session “Amangugu” intervention over a 6- to 8-week period. Intervention has three main aims: increasing maternal HIV disclosure; to increase children’s knowledge about HIV and health; to improve the quality of custody planning for children with HIV-positive mothers	Pre- and post-data collection, including qualitative measures, were completed for all mothers irrespective of the level of disclosure they achieved	Maternal disclosure (partial or full) to the study child following participation in the study was collected at visit 5	All mothers disclosed something to their children: 11/24 disclosed fully using the words “HIV” while 13/24 disclosed partially using the word “virus”

Rochat et al. (39)	281 HIV-infected women and their HIV-uninfected children aged 6–10 years in South Africa	Uncontrolled pre and post-intervention evaluation	Each mother served as her own control	The “Amagugu” intervention includes six structured counseling sessions conducted with the mother at home but no direct intervention with children. There were two intervention stages: a pre-disclosure stage when the counselor worked with the mother to prepare and train her toward disclosure, and a post-disclosure stage, when the mother was counseled on health promotion and custody planning	Pre- and post-intervention evaluation; In addition to baseline and post-intervention, maternal and child mental health was assessed using the General Health Questionnaire (GHQ12) and the Child Behavior Checklist	The primary outcome of this research was disclosure (full, partial, none) and secondary outcomes included maternal and child mental health	171 (60%) women “fully” disclosed and 110 (40%) women “partially” disclosed their HIV status to their child. Women who perceived their health to be excellent were less likely to “fully” disclose compared to those considering their health to be poorer [adjusted odds ratio 0.48 (0.28–0.95), *P* = 0.11]. Compared to those not in a current partnership, those with a current partner were almost three times more likely to “fully” disclose [adjusted odds ratio 2.92 (1.33–6.40), *P* = 0.008]

Simoni et al. (40)	20 HIV-positive outpatients with at least one child who was unaware of the parent’s HIV status in Shanghai, China	RCT with blinded assessment	Control condition (*n* = 10) received treatment as usual	IG received three counseling sessions for up to 1 h per session over 4 weeks in addition to standard care. The intervention (based on Chinese Parental HIV Disclosure Model) has three components: decision-making, the disclosure event, and related outcomes. Session 1: parents share the story of their diagnosis. Session 2: provision of psycho-education of what parents should expect during disclosure. Session 3: parents develop a plan for achieving their desired position along the disclosure continuum	Baseline, immediate post-intervention (4 weeks), and follow-up (13 weeks). Patients were given 1-h paper-based assessment survey. Participants in both conditions were reimbursed 150 RMB (~$25) for completing each session	Disclosure distress (3 item questions, numerical response ranging from 1 to 4), disclosure self-efficacy (2 item questions, numerical response 1–4), and disclosure behaviors (continuum ranging from 0 = no disclosure to 6 = full disclosure and open communication about HIV)	Participants in the intervention arm indicated a sharp decrease in level of disclosure distress from baseline to follow-up (OR = 0.17, 95% CI: 0.03–0.91). Disclosure self-efficacy improved significantly for the IG than the control group from baseline to follow-up (OR = 9.00, 95% CI: 2.06–39.29). Participants in the IG reported significantly greater movement along the disclosure behavior continuum than those in the control from baseline to post-intervention (β = 1.40, 95% CI: 0.31–2.50)

## Results

### Inclusion and Exclusion of Studies

**Figure 1:**
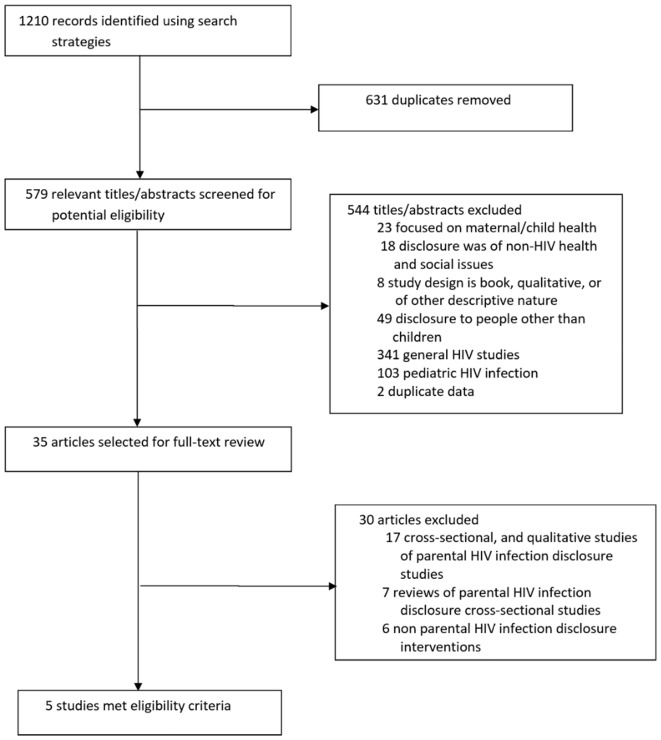
Results of literature search. Summary of search results identifying potentially relevant, screened, and selected articles.

The electronic database searches retrieved 1,210 records (172 from PsycInfo, 184 from Global Health, 313 from Web of Science, 273 from Embase, 268 from PubMed). After removing the duplicates in RefWorks, 579 records were screened (Figure 1). Of these, 544 were excluded because they were mostly general HIV studies examining prevention of mother-to-child transmission, treatment adherence, etc. Thirty-five records were selected at the abstract level for full text review because they described a study focusing on parental HIV infection. Thirty of the 35 were excluded because they were cross-sectional or qualitative studies of parental HIV disclosure, and systematic reviews.

The final sample consisted of five studies published between 2001 and 2015, all of which evaluated interventions designed to promote parental HIV serostatus disclosure to children. The children in two of the studies (38, 39) were HIV-negative, and the remaining three studies (36, 37, 40) did not specify the HIV status of the children. The sample size for these studies ranged from 20 families (40) to 307 families (36). Two of the studies were conducted in South Africa (38, 39), two in the United States (36, 37), and one in China (40).

### Intervention Theoretical Framework and Design

Four of the five studies selected for this review included a theoretically informed intervention to improve parental HIV serostatus disclosure to their children. Rotheram-Borus et al. (36) employed social learning theory, which provides a framework for how individuals can change their behavior while highlighting a set of factors that must be changed, such as skills, expectations of competence and efficacy, and ability to express and control one’s feelings. Rochat et al. (38, 39) developed the Amagugu intervention based on the Model of HIV-Disclosure Decision Making (MHDDM). One of the key characteristics of MHDDM is the encouragement of individuals to consider potential benefits and risks of disclosure while allowing for different types and levels of disclosure. Murphy et al. (37) also relied on MHDDM to guide the development of the Teaching, Raising, and Communicating with Kids (TRACK) Program. Simoni et al. (40) drew from the Disclosure Process Model of Chaudoir et al. (30), the model for maternal HIV disclosure from Murphy et al. (37) and qualitative interviews with PLWH, community advisory board members, and HIV care providers to develop the Chinese Parental HIV Disclosure Model. The model comprises of three main components which include decision-making, the disclosure event, and related outcomes.

Three of the studies used two-arm randomized controlled trials (RCTs), in which the intervention group (IG) was given enhanced care while the control group received standard care (36, 37, 40). The other studies used an uncontrolled pre- and post-intervention evaluation (38, 39).

### Intervention Content

Rotheram-Borus et al. (36) implemented a 24-session intervention for the participants in the IG over 12 Saturdays in small groups. The intervention was divided into two modules, and two sessions were held each Saturday; the first module was delivered to parents alone for four Saturdays, and the second module to both parents and their adolescents for eight Saturdays. Module 1 focused on parents’ adaptation to living with HIV, how to cope with the health effects of HIV, opportunities for disclosure to children, and plans to help children cope with the diagnosis. In module 2, parents learned to initiate custody plans, reduce risk behaviors, create, and maintain positive family routines. Adolescents in module 2 focused on healthy adaptation to their parents’ illness, worked to improve parent–youth relationships, and learned ways to reduce youths’ risk behaviors. The Amagugu intervention implemented by Rochat et al. (38, 39) included printed materials, therapeutic tools, and child-friendly activities and games on HIV disclosure. It was delivered in six structured counseling sessions, with each session having specific contents, activities, and materials. The sessions included topics on positive parenting, positive families, positive life stories, positive practices, positive planning, and positive futures. As there was no direct intervention with children, mothers were supported to disclose independently.

The intervention by Murphy et al. (37) included three sessions. The first session addressed children’s typical development, *pros and cons* of disclosure, and family routines as a foundation for disclosure. The second session focused on mother–child communication and provided quotes from mothers and children on disclosure. The last session utilized roleplaying to practice disclosure, during which the facilitator also provided positive reinforcement. The intervention conducted by Simoni et al. (40) was also composed of three sessions. The first session involved a discussion on the advantages and disadvantages of disclosure, and the provision of instructional information on useful family communication skills. The second session addressed what the parents can expect from their children during disclosure. During the third session, parents created a plan that would help them reach their goal on the disclosure continuum (0—no disclosure to 6—complete disclosure).

### Disclosure Measurement

Disclosure was measured differently across the studies. In the study conducted by Rotheram-Borus et al. (36), presence and absence of disclosure to all adolescents in the family was recorded numerically, with 1 for presence and 0 for absence. Studies by Rochat et al. (38, 39) considered all levels of disclosure, including partial (i.e., explaining that the mother has a “virus”), full (i.e., the mother used the term “HIV”), and no disclosure. Murphy et al. (37) asked mothers if they had disclosed to the child at each follow-up. If she had not disclosed, baseline information was used again. If she had disclosed, the following information on disclosure was collected: date of disclosure, the child’s reaction to disclosure, and how the mother felt she handled the disclosure. Simoni et al. (40) measured disclosure behaviors by using a visual Disclosure Behaviors Continuum, which ranged from 0 for no disclosure to 6 for full disclosure and open communication about HIV.

### Summary of Study Findings

The findings of the interventions were mixed across the studies. Murphy et al. (37) found that mothers in the IG were more likely (OR = 6.33, 95% CI: 1.64–24.45) to disclose their HIV status than those in the control group, with 33% disclosing in the IG compared with 7.3% in the control group. Rotheram-Borus et al. (36) found no statistically significant difference in disclosure across conditions. In the pilot study by Rochat et al. (38), all mothers disclosed to their children with a varied level of disclosure, with 11 of 24 mothers reporting full disclosure by using the word “HIV” while the remaining 13 mothers disclosed partially using the word “virus.” In the follow-up study conducted by Rochat et al. (39), about 60% of the mothers (*n* = 171) disclosed fully and 40% (*n* = 110) partially disclosed their HIV status to their child. They also found that mothers who reported excellent health were less likely (OR = 0.48, 95 CI: 0.28–0.95) to fully disclose their status than those who reported poorer health. In addition, mothers who were in a relationship were more likely (OR = 2.92, 95% CI: 1.33–6.40) to disclose their relationship fully than those who were not in a current partnership. In the study conducted by Simoni et al. (40), participants in the IG reported statistically significant greater movement along the Disclosure Behaviors Continuum than those in the control from baseline to post-intervention (β = 1.40, 95% CI: 0.31–2.50), with the intervention parents moving on average from 1.2 to 3.0, and the TAU parents from 0.7 to 1.4.

## Discussion

The objective of this paper was to contribute to the growing literature on interventions for parental HIV serostatus disclosure. We systematically reviewed the literature and found a small number of studies have evaluated such interventions. The findings of the interventions were mixed, with four (37–40) of the studies reporting an increase in parental HIV disclosure. Although there was no geographical limit for the search, the location of the studies that met the inclusion criteria for this review were the United States, China, and South Africa. The cultural differences in the settings where the interventions were conducted may also influence if, and how parents disclose their status to their children. Disclosure of HIV status is behavioral in nature and driven by many contextual issues including culture. For example, the barriers and cultural norms that prevent open communication about sexuality and HIV/AIDS between parents and children in sub-Saharan Africa (41, 42) may not be present in the United States. However, in sub-Saharan Africa, the desire to disclose knowledge of a HIV-positive status is perhaps a consequence of growing up in a society where there are frequent reminders that identities are relational and that this rationality is vital for necessary support with living with HIV. Disclosure occurred because parents naturally belonged to, or are part of, particular familial, local, or ethnic groups, whereby illness was viewed as a responsibility of the collective (20).

Additionally, three of the studies included only mothers living with HIV (37–39). The predominance of women in parental disclosure interventions to an extent may reflect the gender proportions of the adult population infected with HIV globally (43). Since child care is often the sole responsibilities of mothers, it was not uncommon for some of the mothers to fully disclose their status following knowledge of their sero-positive status (20). The generally held view that mothers are expected to provide emotional care and support for their children or family members even in the context of HIV disclosure (20) was evident in the interventions involving mothers. Another interesting finding is that mothers who were in a relationship were more likely to disclose to their children than those who were not (39). A potential explanation for this finding may be that parental HIV disclosure to children becomes easier once parents have disclosed their HIV status to a primary sexual partner, hence providing support for parental HIV disclosure interventions to have a disclosure to sexual partner component (44).

Similar to other studies, parental HIV disclosure was associated with positive mental health outcomes for the children in the IG (14). For example, Rochat et al. (39) found a significant decrease in anxious-depressed, withdrawn-depressed, aggressive behavior, and rule-breaking syndromes among children. However, the decrease in withdrawn-depressed syndrome after the intervention was larger among children of mothers who partially disclosed than those who fully disclosed, indicating the importance of gradual disclosure or partial disclosure. Similarly, Murphy et al. (37) reported a reduction in depression and anxiety among children of mothers in the IG. Rotheram-Borus et al. (36) also reported lower levels of emotional distress, of multiple problem behaviors, of conduct problems, and of family-related stressors. These findings suggest that parental HIV disclosure can be beneficial for children when parents receive the proper training and guidance on how, and when to disclose to their children. In contrast, unintentional and poorly prepared parental HIV disclosure can have detrimental effects on children (1). The benefits observed among children in the IG may be the results of other areas addressed by the intervention such as parenting skills and communication with children (39). The ability for parents and children to openly communicate about HIV and other concerns with their children may improve parent–child relationship and children’s coping behaviors (45).

While most of the interventions included in this review for parental HIV disclosure offer promising findings, there are a number of limitations from the existing literature that need to be addressed in future studies. First, only two of the interventions included fathers. The other three focused only on mothers. The lack of disclosure interventions for HIV-positive fathers reflects the broad HIV literature as HIV-positive men are overlooked and understudied (46). Research indicates that notions of male identity, family, and community influence disclosure among HIV-positive men (19). However, little research has examined how fatherhood affects men’s experiences with their HIV status, especially in the context of parental disclosure (47). Future studies should include HIV-positive fathers or both parents, if available, in order to understand their approach to parental disclosure and address their needs. Second, the South African studies were unable to directly interview the children due to ethical reasons. More efforts are needed to provide approval for studies conducted in developing countries to include children in order to better assess the effect of the intervention on their mental health. Third, more rigorous studies are needed to determine the best practices for parental disclosure as only three of the included interventions were RCTs, with one of the studies having a sample size of 20 (40). The three successful RCTs provide the foundation for future studies to adapt the materials for interventions in different cultural settings such as Europe, Asia, the Caribbean, and other African countries. Fourth, none of the studies focused on immigrants. Immigrants affected by HIV face additional challenges with parental disclosure such as legal status and geographical separation from family and are in need of culturally adapted strategies to help them with the disclosure process to their children (34, 48). Finally, the individual studies meeting the inclusion criteria were limited by a number of methodological issues, including sampling and disclosure measurements used by different interventions.

Non-disclosure of personal health information has been shown to be unhealthy (49). Likewise, research indicates that lack of disclosure may negatively affect parents’ health. For example, parents who have not disclosed their HIV status have reported skipping medication and medical appointments in order to prevent their children from becoming aware of their ill health (14). The health benefits of disclosure can extend beyond the parents as findings from the studies suggest the children in the IG have better mental health outcomes than those in the control group due to the possible improvement in parent–child communication and relationship. Therefore, it is crucial for healthcare professionals to receive training on how to facilitate parents to make informed choices about disclosure and provide the tools and resources to disclose when they are ready. These resources can include printed materials and child-friendly activities and games (39) that parents can use to help them move along the disclosure behavior continuum (40).

Despite these potential benefits, parental HIV disclosure remains challenging and many parents may never disclose. More evidence-based interventions are needed to help parents facilitate and manage the parental disclosure process. As more people continue to live longer with HIV and desire to have children (50–52), more parents will be faced with the decision to disclose to their children. Overall, this review has identified intervention strategies that have proven to be efficacious in improving parental HIV disclosure and can be modified to encourage and support parents in different cultural contexts with the difficult process of disclosing to their children.

## Author Contributions

All authors are in agreement regarding the content of the article. All authors have contributed to the conceptualization, design, and analysis, and all were involved in drafting and reviewing the article. DC took overall responsibility for the conceptualization and design of the review, collating the articles, analyzing the data, and writing the article. MT, JI, and SM were involved in conceptualization and design of the review as well as writing and editing the article. LH and GS searched for the articles in the review, assessed them for relevance, and were involved in writing, reviewing, and editing the final article.

## Conflict of Interest Statement

The authors declare that the research was conducted in the absence of any commercial or financial relationships that could be construed as a potential conflict of interest.
